# Physician Altruism and Spending, Hospital Admissions, and Emergency Department Visits

**DOI:** 10.1001/jamahealthforum.2024.3383

**Published:** 2024-10-11

**Authors:** Lawrence P. Casalino, Shachar Kariv, Daniel Markovits, Raymond Fisman, Jing Li

**Affiliations:** 1 Deptartment of Population Health Sciences, Weill Cornell Medical College, New York, New York; 2 Department of Economics, University of California, Berkeley; 3 Yale Law School, New Haven, Connecticut; 4 Department of Economics, Boston University, Boston, Massachusetts; 5 Department of Pharmacy, University of Washington, Seattle

## Abstract

**Importance:**

Altruism—putting the patient first—is a fundamental component of physician professionalism. Evidence is lacking about the relationship between physician altruism, care quality, and spending.

**Objective:**

To determine whether there is a relationship between physician altruism, measures of quality, and spending, hypothesizing that altruistic physicians have better results.

**Design, Setting, and Participants:**

This cross-sectional study that used a validated economic experiment to measure altruism was carried out between October 2018 and November 2019 using a nationwide sample of US primary care physicians and cardiologists. Altruism data were linked to 2019 Medicare claims and multivariable regressions were used to examine the relationship between altruism and quality and spending measures. Overall, 250 physicians in 43 medical practices that varied in size, location, and ownership, and 7626 Medicare fee-for-service beneficiaries attributed to the physicians were included. The analysis was conducted from April 2022 to August 2024.

**Exposure:**

Physicians completed a widely used modified dictator-game style web-based experiment; based on their responses, they were categorized as more or less altruistic.

**Main Measures:**

Potentially preventable hospital admissions, potentially preventable emergency department visits, and Medicare spending.

**Results:**

In all, 1599 beneficiaries (21%) were attributed to the 45 physicians (18%) categorized as altruistic and 6027 patients were attributed to the 205 physicians not categorized as altruistic. Adjusting for patient, physician, and practice characteristics, patients of altruistic physicians had a lower likelihood of any potentially preventable admission (odds ratio [OR], 0.60; 95% CI, 0.38-0.97; *P* = .03) and any potentially preventable emergency department visit (OR, 0.64; CI, 0.43-0.94; *P* = .02). Adjusted spending was 9.26% lower (95% CI, −16.24% to −2.27%; *P* = .01).

**Conclusions and Relevance:**

This cross-sectional study found that Medicare patients treated by altruistic physicians had fewer potentially preventable hospitalizations and emergency department visits and lower spending. Policymakers and leaders of hospitals, medical practices, and medical schools may want to consider creating incentives, organizational structures, and cultures that may increase, or at least do not decrease, physician altruism. Further research should seek to identify these and other modifiable factors, such as physician selection and training, that may shape physician altruism. Research could also analyze the relationship between altruism and quality and spending in additional medical practices, specialties, and countries, and use additional measures of quality and of patient experience.

## Key Points

**Question:**

Is there an association between physician altruism (which may indicate intrinsic motivation to act professionally by putting patients first) and care quality and spending?

**Findings:**

In this cross-sectional study that linked Medicare claims to data from a validated economic experiment, patients of altruistic physicians were significantly less likely to have a potentially preventable hospital admission or potentially preventable emergency department visit, and had 9% lower health care spending.

**Meaning:**

In an era of increasing attempts to use financial incentives to influence physicians’ behavior, hospitals, medical practices, medical schools, and policymakers may want to consider seeking to increase, or at least preserve, physician altruism.

## Teaser

This observational study examines the association between physician altruism, measures of quality, and spending, hypothesizing that altruistic physicians have better results.

## Introduction

Complex incentives in the health care system confront physicians with trade-offs between their own self-interest and patient interests.^1^ Physicians must, for example, frequently decide whether to recommend medical services that are profitable for the physician but may only marginally benefit patients,^2^ and whether to spend extra time with socioeconomically disadvantaged patients who may face barriers to understanding and gaining access to the care they need.^3,4,5,6,7^

Professional ethics have traditionally been embraced as a guide to help physicians make such choices and altruism—putting the patient first—has long been considered a fundamental component of physician professionalism.^8,9,10,11^ For example, the American Board of Internal Medicine stated that “Altruism is the essence of professionalism. The best interest of patients, not self-interest, is the rule.”^12^ However, little is known empirically about the extent to which physicians behave altruistically, and nothing is known about whether and how physician altruism is related to the quality of health care.

In prior work, using an economic experiment we conducted on a nationwide convenience sample of primary care physicians and cardiologists, we found that physicians on average were more altruistic than the general population and than an elite sample of highly educated, high-earning individuals.^13^ Herein we report results of what we believe to be the first empirical, exploratory study of the relationship between physician altruism and measures of health care quality and spending. Our primary hypothesis was that patients of altruistic physicians would have better quality and lower health care spending as measured using Medicare fee-for-service claims data.

## Methods

### Overview and Data Sources

In prior research, we measured altruism in 2019 among a nationwide sample of physicians using an economic experiment.^13^ For the research presented here, we used a 20% sample of fee-for-service Medicare Parts A and B claims data from 2019 to construct measures of quality and spending for patients attributed to the sampled physicians. We merged the experimental data, claims data, and physician responses to a brief survey that accompanied the experiment, and used multivariable regressions to analyze the relationship between physician altruism and measures of quality and spending. The study analysis plan is available in Supplement 1. In prespecified secondary analyses, we analyzed this relationship by physician specialty and for patient subgroups, and the relationship between altruism and time spent on patient care.

The institutional review board of the Weill Cornell Medical College reviewed and approved this study. Written informed consent was obtained from all participants. This study followed the Strengthening the Reporting of Observational Studies in Epidemiology (STROBE) reporting guidelines.

### Experimental Design

We adopted a widely used modified dictator-game style web-based experiment to measure the altruism of physician participants. There is no gold standard for measuring altruism, so these experiments cannot be directly validated; validation is done by measuring the success of the experiments in predicting actual behavior.^14,15,16,17,18,19,20,21,22,23^ Research using this design has been published in *Science* and leading economics journals, and been shown, for example, to predict voting behavior^23^ and medical student plans to train in lower vs higher income specialties and in underserved areas.^17^

The experiment, which required approximately 15 minutes on the physician’s computer, asked physicians to allocate real money between themselves and an anonymous other person drawn randomly from the Understanding America Study (UAS) panel, which is broadly representative of the US population.^24^ Each allocation decision was represented as a budget line in a 2-dimensional graphical interface, where each point represented a possible allocation, with y- and x-coordinates representing the payoff to the physician and the other person (eMethods 1 in Supplement 2 provides details). The y-intercept represented the maximum possible amount allocable to self (the physician, with the other receiving zero), and the x-intercept represented the maximum possible amount allocable to other (the physician receiving zero).

The Web interface randomly generated 25 budget lines with varying intercepts, and the physician made allocation decisions on each line. As is customary in similar experiments, 1 of the 25 decisions was randomly selected,^14^ and the participant and the person paired with the participant each received real money corresponding to the amount allocated during the selected round. The maximum possible payoff was $250, with an average payoff of $156 for physicians who chose to allocate everything to themselves.

Physicians were unaware that the study focused on altruism (it was framed as a study of physician decision-making), and understood that they would not receive a penalty for allocating the maximum possible amount to themselves nor a benefit from allocating money to the other person (the recipient was unknown and allocations remained anonymous). Money allocated to the other therefore suggests altruism (ie, concern for others).

### Study Population

We focused on physicians in primary care (internal medicine and family practice) and cardiology, one of the most common subspecialties. These 2 specialties represent a wide income range, which might be associated with differences in altruism.^18^ To recruit physician participants, between October 2018 and November 2019 we contacted leaders of primary care and cardiology practices and multispecialty practices that included primary care physicians and/or cardiologists. There is no generally accepted national database of medical practices, so we identified practices in 3 ways: (1) via practice leaders known to 1 of the authors (L.C.), (2) via practice leaders referred by one of the author’s (L.C.) contacts, and (3) via web searches. Leaders of practices were asked to fill out a survey with questions on practice characteristics and to forward to their physicians an invitation to participate in a study of physician decision-making. The study was framed as being about physician decision-making; altruism was not mentioned. The invitation contained a link to participate in the online experiment and a brief physician survey (eMethods 2 in Supplement 2 provides recruitment details; eMethods 3 and 4 in Supplement 2 include the survey questionnaires).

We attributed Medicare beneficiaries to the physician who, regardless of specialty, provided the plurality of evaluation and management claims for the beneficiary in 2019. Physicians were included in the analysis if they completed the experiment and survey and had at least 3 attributed Medicare beneficiaries (eFigure 1 in Supplement 2 provides the sample flowchart).

### Measures

#### Independent Variable

The independent variable was physician altruism. We measured the degree of altruism as the relative weight that the physician placed on their own payoff vs the payoff to the other person in making allocation decisions. The altruism parameter (α) thus ranged from 0 to 1; lower values indicate higher altruism. To derive this parameter, we estimated a separate model for each physician assuming that they maximized a constant elasticity of substitution (CES) utility function commonly used in empirical demand analysis in economics, using data on the allocations they made for all 25 rounds of decisions; this measure has been used for similar experiments in prior literature.^13,14,17^

In our primary analyses, we used a dichotomous version of this altruism parameter α, classifying a physician as altruistic if we rejected that their α equaled 0.5 against the alternative hypothesis that α was lower than 0.5, using a 1-sided *t* test at 5% significance level. This indicated that the physician placed more weight on the other’s payoff than their own. In 3 sensitivity analyses, we (1) classified physicians as altruistic if their α point estimate was lower than 0.5, (2) used a 1% instead of 5% significance level to test the hypothesis of whether a given estimated α equals 0.5, and (3) used a continuous measure of α. Measure 1 was a less conservative measure of altruism and classified a higher number of physicians as altruistic (eTable 1 in Supplement 2); measure 2 was a stringent test of the association between altruism and the dependent variables; measure 3 imposed the restriction that the association between α and outcomes be linear.

#### Covariates

Covariates included physician characteristics (age, sex, specialty) from the physician survey responses, medical practice characteristics (size, ownership) from the practice leader survey responses, and patient characteristics (sex, age, race and ethnicity, Centers for Medicare & Medicaid Services [CMS]−hierarchical condition category [HCC] risk score, dual-eligibility for Medicaid and Medicare); demographic characteristics were collected from the Medicare Beneficiary Summary File. Race and ethnicity and dual-eligibility were included given the widespread interest in potential disparities in care. Dual-eligible patients are eligible for both Medicaid and Medicare. The race and ethnicity categories came from the Medicare Beneficiary Summary File’s Research Triangle Institute Code variable; “other” race includes Asian or Pacific Islander, American Indian or Alaska Native, and any additional race and ethnicity category not specified.

#### Dependent Variables

We used Medicare claims to construct measures of quality and spending as primary dependent variables. For quality we measured (1) whether the patient had any ambulatory care–sensitive admission (ACSA) and (2) whether they had any ambulatory care–sensitive emergency department (ACSED) visits in 2019. These measures were derived based on the algorithm published by the Agency for Healthcare Research and Quality.^25^ They are widely used and represent adverse events that could potentially be prevented by high-quality outpatient care.^26,27^ We refer to these as *quality* measures, acknowledging that they do not cover all areas of quality, and we refer to these measures and spending collectively as *outcomes*. We calculated spending in 2019 by summing Medicare allowed amounts (including Medicare, patient, and third-party payments) paid across all Medicare Parts A and B services for a given patient. We geographically adjusted all spending measures using the county-specific actual-to-standardized cost ratios published by CMS. We used the logged value of spending measures, which is less sensitive to outliers.

As secondary dependent variables, we examined self-reported time spent on patient care (a potential mechanism through which altruism may be linked to quality and spending), focusing on 2 measures: the number of patients a physician reported seeing in a typical 3-hour period, from which information on time spent with each patient can be inferred, and time spent per day on patient care at home. We hypothesized that altruistic physicians would spend (1) more time per patient (since this may lead to better quality but less income for the physician) and (2) more time at home on patient care. Since the latter was highly skewed, we used a dichotomous measure of whether the physician spent at least 1 hour on patient care at home.

### Statistical Analyses

We used multivariable regressions to examine associations between being attributed to an altruistic physician and the probability of having any ACSA or ACSED, as well as per patient spending. We used logistic regressions for quality variables, log-linear regression models for spending, and ordinary least squares for dependent variables indicating time spent on patient care. Analyses were conducted for all physicians and separately for primary care physicians and for cardiologists. All regressions controlled for physician, patient, and practice characteristics, with standard errors clustered by physician. All statistical tests (except those used to classify the altruism category) were 2-sided with 5% significance level. We performed multiple hypothesis testing adjustments on our primary analyses using the Holm-Bonferroni method.^27,28,29^ Analyses was conducted from April 2022 to August 2024 using Stata/MP statistical software, version 16 (StataCorp, LLC).

In secondary analyses, we explored the association of altruism with outcomes for dual Medicare/Medicaid eligible vs nondual patients, patients of White race compared with those of any other race or ethnicity, and patients in the highest quintile of risk vs other patients. We hypothesized that altruistic physicians would have better outcomes than other physicians for patients who may require extra time and effort (often not reimbursed by Medicare) because they are medically complex and/or socioeconomically disadvantaged.^5,30,31^ We also explored the association between altruism and physician time per patient visit and time spent at home on patient care.

## Results

Of the 87 contacted practices, 43 (49.4%) participated (eMethods 2 in Supplement 2). Eleven participating practices were known to the authors, 25 referred by the authors’ contacts, and 7 identified via web searches aimed at creating a geographically diverse sample of practices. There was no significant difference in altruism based on the method by which the practices were identified (eTable 2 in Supplement 2).

The study population included 7626 Medicare patients attributed to 250 physicians, all of whom completed the survey (eFigure 1 in Supplement 2). The mean (SD) patient age was 76 (7) years; 4259 (56%) were women. Overall, 1599 patients (21%) were attributed to 45 physicians (18%) classified as altruistic (Table 1). These patients were similar to patients attributed to nonaltruistic physicians. Altruistic and nonaltruistic physicians were similar except that those classified as altruistic were more likely to work in practices with fewer than 36 physicians (24% for altruistic vs 12% for nonaltruistic, *P* = .03). There was no significant difference in altruism by physician specialty. eFigure 2 in Supplement 2 shows the distribution of point estimates for physician altruism using our primary method for categorizing physicians as altruistic; lower values of α imply higher altruism.

**Table 1.** Summary Statistics of Physician and Patient Characteristics

Variable	No. (%)	
	Not altruistic^a^	Altruistic
**Physician characteristics**		
No. of physicians	205	45
Sex		
Female	78 (38)	18 (40)
Male	127 (62)	27 (60)
Age group, y		
≤39	57 (27.8)	12 (26.7)
40-49	67 (32.7)	14 (31.1)
50-59	45 (22.0)	13 (28.9)
≥60	36 (17.6)	6 (13.3)
Specialty		
Internal medicine	100 (48.8)	22 (48.9)
Family medicine	34 (16.6)	10 (22.2)
Cardiology	71 (34.6)	13 (28.9)
Private practice	50 (24.4)	16 (35.6)
Practice size		
<36	24 (11.7)	11 (24.4)
36-100	73 (35.6)	14 (31.1)
101-350	35 (17.1)	7 (15.6)
350-1600	73 (35.6)	13 (28.9)
**Patient characteristics**		
No. of attributed patients	6027	1599
Patient sex		
Female	3390 (56.2)	869 (54.3)
Male	2436 (43.8)	730 (45.7)
Age, mean (SD), y	76.4 (7.0)	76.6 (7.1)
Race and ethnicity^b^		
Black	581 (9.6)	155 (9.7)
Hispanic	225 (3.7)	50 (3.1)
White	4782 (79.3)	1312 (82.1)
Other	264 (4.4)	47 (2.9)
Unknown	175 (2.9)	35 (2.2)
CMS-HCC score	1.2 (1.0)	1.2 (1.0)
Dual-eligible^b^	386 (6.4)	89 (5.6)

Abbreviation: CMS-HCC, Centers for Medicare & Medicaid Services-Hierarchical Condition Category.

^a^ Physicians were classified as altruistic if we could reject that H_0_: α = 0.5 vs H_1_: α < 0.5 using a 1-sided *t* test at 5% significance level, and nonaltruistic otherwise.

^b^ Race and ethnicity and dual-eligibility were included because of widespread interest in potential disparities in care. Dual eligible patients are eligible for both Medicaid and Medicare. The race and ethnicity categories come from the Medicare Beneficiary Summary File’s Research Triangle Institute (RTI) Code variable. The other category includes Asian/Pacific Islander, American Indian/Alaska Native, and any additional race and ethnicity categories not specified (these are classified as “other” in the RTI race variable).

In unadjusted analyses, 34 patients (2.1%) attributed to altruistic physicians had any ACSA compared with 156 patients (2.6%) of other physicians (*P* = .32); 47 patients (2.9%) attributed to altruistic physicians had any ACSED compared with 221 (3.7%) for other patients (*P* = .11) (Table 2). Annual spending for patients of altruistic physicians was $9514 vs $8903 for other patients (*P* = .20).

**Table 2.** Associations Between Physician Altruism and Quality and Spending^a^

Outcome variable	Unadjusted mean		Adjusted association, odds ratio for quality of care; % difference for spending (95% CI)^c^	*P* value
	Patients attributed to nonaltruistic physicians (n = 6027)^b^	Patients attributed to altruistic physicians (n = 1599)		
**Quality of care**				
Any ambulatory care sensitive admission				
All physicians	0.03	0.02	0.60 (0.38 to 0.97)	.04
Primary care	0.02	0.02	0.88 (0.53 to 1.44)	.59
Cardiology	0.05	0.02	0.31 (0.14 to 0.66)	.003
Any ambulatory care sensitive emergency department visit				
All physicians	0.04	0.03	0.63 (0.43 to 0.94)	.02
Primary care	0.03	0.02	0.60 (0.37 to 0.99)	.04
Cardiology	0.06	0.04	0.40 (0.21 to 0.78)	.007
**Total spending, $**				
All physicians	8903	9514	−9.3 (−16.2 to −2.3)	.01
Primary care	7994	9129	−5.3 (−12.9 to 2.2)	.17
Cardiology	12 517	10 231	−17.3 (−36.3 to 1.8)	.08

^a^ Physicians were classified as altruistic if we could reject that H_0_: α = 0.5 vs H_1_: α < 0.5, using a 1-sided *t* test at 5% significance level, and nonaltruistic otherwise.

^b^ Unadjusted numbers for any ambulatory care–sensitive admissions and any ambulatory care–sensitive emergency department visits reflect proportion of patients with those outcomes.

^c^ Adjusted associations were estimated using multivariable regressions (logistic regressions for quality of care measures and log-linear model for spending) that controlled for all physician, practice and patient characteristics in Table 1. Each row reports results from a separate regression for all physicians or by specialty (primary care and cardiology). Standard errors were clustered by physician.

In our primary analyses, adjusted for physician, patient, and practice characteristics, patients attributed to altruistic physicians were much less likely to have any ACSA (odds ratio [OR], 0.60; 95% CI, 0.38-0.97; *P* = .03) or any ACSED (OR, 0.64; 95% CI, 0.43-0.94; *P* = .02) (Table 2). These represent a 38% reduction in ACSAs (an absolute reduction of 1 percentage point compared with the 2.6 percentage point mean for patients of nonaltruistic physicians) and a 41% reduction in ACSEDs (an absolute reduction of 1.5 percentage points compared to a mean of 3.7 percentage points), respectively (eMethods 5 in Supplement 2). Contrary to the unadjusted result, adjusted spending was 9.3% lower (95% CI, −16.2% to −2.27%; *P* = .01). Results stratified by specialty were qualitatively similar. All significant results were robust to the Holm-Bonferroni multiple hypothesis testing adjustments except for the ACSED results for primary care physicians (eMethods 6 in Supplement 2).

In our first sensitivity analysis using alternative methods of classifying physicians as altruistic, 85 physicians (34%) were classified as altruistic using a cutoff of 0.5 (eTable 1 in Supplement 2), almost doubling the number classified as altruistic using our primary definition. Using this broader, less conservative measure of altruism, 2 of 3 point estimates for outcomes were in the same direction as our primary analysis (the OR for any ACSED was 1.0), but not statistically significant (eFigure 3 in Supplement 2). In our second sensitivity analysis, using 0.01 rather than 0.05 to test significance, all 3 point estimates were similar to our primary analysis, although only the spending estimate was statistically significant. In our third sensitivity analysis, measuring altruism as a continuous variable, all 3 point estimates for outcomes were very similar to the point estimates in our primary analysis, although results did not reach statistical significance.

In secondary analyses of patient subgroups by Medicaid eligibility, race, and risk score, the point estimates for 17 of the 18 associations between physician altruism and outcomes were in the same direction as in our primary analyses (lower than 1, suggesting better quality; Figure), although only 6 estimates were statistically significant, possibly because of the smaller sample sizes in these subgroups.

**Figure.**
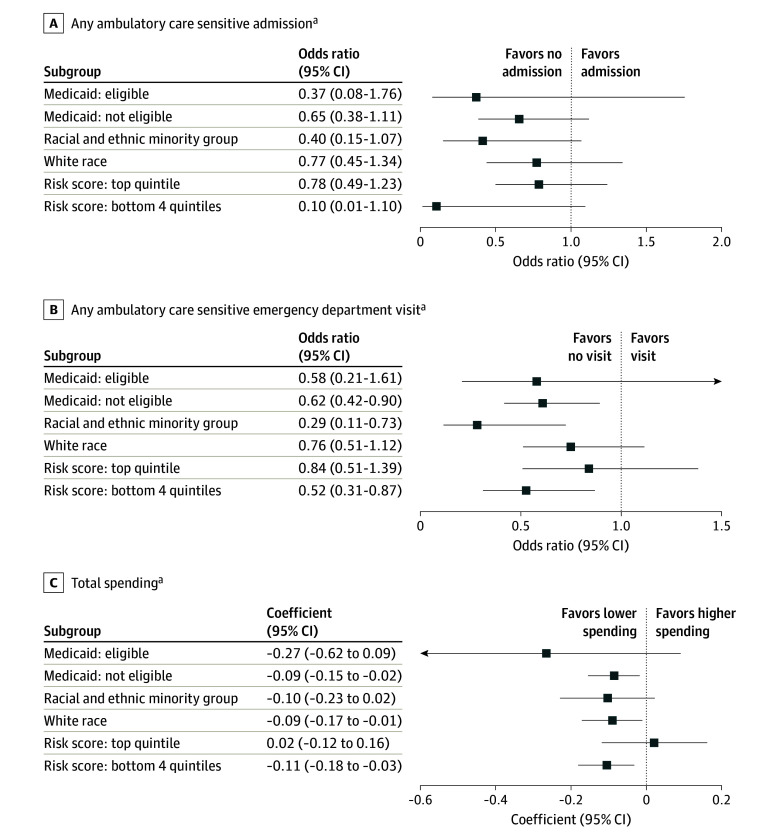
Subgroup Analyses of Total Spending and of Potentially Preventable Hospital Admissions and Emergency Department Visits ^a^Each coefficient and the associated 95% CIs were estimated from a separate regression analogous to those in Table 2, in a patient subgroup. Patients in the top quintile of risk score were the 20% of patients in our sample with the highest Centers for Medicare & Medicaid Services-Hierarchical Condition Category risk scores and were those with the highest disease severity.

In most supplementary analyses, altruistic physicians reported spending more time per patient visit and more time at home on patient care, although most results were not statistically significant (eTables 3-6 in Supplement 2).

## Discussion

We found that experimentally measured physician altruism was significantly associated with outcomes for patients, including 38% fewer ambulatory care sensitive admissions, 41% fewer potentially preventable emergency department visits, and 9% ($800) lower spending. Results were qualitatively similar for patient subgroups.

Patients of altruistic physicians might have better outcomes because their physicians choose the most appropriate tests and treatments, and/or because altruistic physicians devote more time and energy to their patients. Our findings that altruistic physicians reported spending more time per patient visit and more time at home on patient care may provide some support for this possible mechanism linking altruism to quality and spending.

While the 18% of physicians who were classified as altruistic may seem low, when we applied the same definition to our prior research,^13^ 5% of a sample of the US population were classified as altruistic. Our primary definition of altruism is conservative; physicians not characterized as altruistic may have some degree of altruism.

### Limitations

This exploratory study has limitations. First, the results show cross-sectional associations, not necessarily causal relationships, between physician altruism and outcomes. For example, it is possible that healthier patients may have selected more (or less) altruistic physicians, although the characteristics of patients of altruistic physicians were very similar to other patients, and mean HCC scores were identical. Second, we used a convenience sample of medical practices. However, our sample included a large number of practices from all regions of the US; we found no significant difference in altruism based on the method by which the practices were identified. Third, we focused on primary care physicians and cardiologists; results might not be generalizable to other specialties. Fourth, we included only patients enrolled in traditional Medicare. Fifth, although our primary analysis, which adjusts for patient, physician, and practice characteristics, shows lower spending for patients of altruistic physicians, unadjusted results show somewhat higher spending. Sixth, our measures of quality, although widely used, do not include process measures, patient experience measures, or measures derived from medical chart reviews. Seventh, our results varied somewhat depending on how we categorized physicians as altruistic, although they were qualitatively similar across all 3 categorizations and when using a very strict (1%) statistical significance level in our primary analysis. Eighth, patients of primary care physicians may be healthier on average than those of cardiologists; therefore, it may be more difficult for primary care physicians to substantially lower admissions and spending, given the relatively low rate of admissions and spending for patients of both altruistic and nonaltruistic physicians (Table 2). Finally, the measures of time spent per patient and time spent at home on patient care were self-reported.

It would be useful to understand altruism (and physician professionalism) not as an innate capacity that a physician has or does not have, but as a dependent variable that depends on physicians’ training,^32^ the conditions in which they work,^33^ their organizations’ culture,^34,35,36^ and the financial and nonfinancial incentives they face.^37,38,39,40^ Our results may be particularly relevant at this time, when physicians face increasing administrative complexity,^41,42^ private equity and health insurer ownership of medical practices,^43,44,45^ and emphasis on rewarding or penalizing physicians’ performance on specific measures.^46^ Our results are consistent with arguments that attempts by hospitals, medical practices, Medicare, Medicaid, and private insurers to improve quality and reduce spending should seek ways to increase, rather than decrease, physicians’ intrinsic motivation to put patients’ interests first.^36,37,47,48,49,50,51,52^

## Conclusion

Policymakers and leaders of hospitals, medical practices, and medical schools may want to consider creating incentives, organizational structures, and cultures that seem likely to increase, or at least not decrease, physician altruism. Further research could seek to identify these and other modifiable factors, such as physician selection and training, that may shape physician altruism. Research could also analyze the relationship between altruism and quality and spending in additional medical practices, specialties, and countries, and use additional quality and patient experience measures.

## Notes

Supplement 1. Trial Protocol

Supplement 2. eMethods 1. Experimental Instructions eMethods 2. Recruitment of Physician Subjects eMethods 3. Survey Questionnaire for Physicians eMethods 4. Survey Questionnaire for Medical Practice Leaders eMethods 5. Estimating Percentage Differences from Logistic Regression Results eMethods 6. Multiple Hypothesis Testing Adjustments Using the Holm-Bonferroni Method eMethods 7. Pre-registered Statistical Analysis Plan eFigure 1. Flowchart of analytic sample eFigure 2. Histogram of altruism parameter α, by altruism classification eFigure 3. Associations between altruism and care quality and spending across different altruism classifications eTable 1. Number of physicians and patients in each category of altruism classification, by model specification eTable 2. Associations between recruitment methods and physician altruism eTable 3. Associations between physician altruism and time spent on patient care eTable 4. Associations between physician altruism and time spent on patient care, classifying altruism by rejecting H0: α=0.5 vs. H1: α<0.5 using a one-sided t-test at the 1% level eTable 5. Associations between physician altruism and time spent on patient care, with α=0.5 as the cutoff for altruism eTable 6. Associations between physician altruism and time spent on patient care, with 1-α as the continuous measure of altruism

Supplement 3. Data Sharing Statement
