# Supplementary material for: Physician Altruism and Spending, Hospital Admissions, and Emergency Department Visits
Source: JAMA Health Forum. 2024 Oct 11;5(10):e243383. doi: 10.1001/jamahealthforum.2024.3383 (PMC11581536; doi:10.1001/jamahealthforum.2024.3383)
Supplement: Supplement 1. — Trial Protocol [file jamahealthforum-e243383-s001.pdf]

Statistical Analysis Plan, Updated

February 21, 2023

Note: This document is updated from the pre-specified statistical analysis plan (SAP) dated January 20, 2022 and registered on the Open Science Framework website: https://osf.io/75j8k. This current document shows updates and changes in analyses made from the pre-specified SAP and are consistent with the submitted manuscript. Changes and updates are highlighted in bold. This document also includes a section on power.

- Title: Physician altruism and care quality and spending among Medicare patients
- Funding: Physicians Foundation
- Research team: Lawrence Casalino, **Shachar Kariv, Daniel Markovits**, **Raymond Fisman,** Jing Li
- Research Question(s)
    1. Do more altruistic physicians as measured in a lab experiment provide better quality of care among Medicare patients?
    2. Do more altruistic physicians as measured in a lab experiment incur lower spending on care for Medicare patients?
    3. Does the relationship between physician altruism and care quality/spending differ by patient socioeconomic status and patient risk?

- Methods
    - Data source(s):
        - Primary data collected in 2019 on altruism of a nationwide sample of physicians in Internal Medicine, Cardiology and Family Medicine
        - 2019 Medicare Parts A & B claims data
    - Overview of analysis plan
        - Study sample (inclusion & exclusion restrictions):
            - **Physicians: 250 physicians with complete survey and experimental data, and who had at least three attributed Medicare patients in 2019** based on the attribution rule from CMS Merit-Based Incentive Payment System (by plurality of primary care claims)

        - Patients: **7,626** Medicare patients **aged 66 and above** who were continuously enrolled from January to December 2019 who were attributed to the **250** physicians included in the study.
    - Approach:
        - Multivariable regressions examining the relationships between altruism (independent variable) and quality of care and spending on care (dependent variables)
        - Stratified analysis by physician specialty (primary care vs. cardiology)
        - Stratified analysis by patient subgroup based on race (white vs. non-white), Medicaid eligibility, HCC top 20% risk score
        - Secondary analysis:
            - ❖ Relationship between altruism and average time spent per patient visit, and time spent on patient care at home before or after work

- Variables:
    - Key independent variable: Altruism (Selfless vs. not selfless)
        - **Primary definition of selfless: cannot reject that α=0.5 vs. α<0.5, using one-sided t-test at 5% significance (this definition was adopted as it is the most stringent and most consistent with the inherent notion of altruism—physicians put unambiguously more weight on others' payoff than their own).**
        - **Alternative definitions for sensitivity analyses:**
            - ❖ **α<0.5 using point estimate only**
            - ❖ **α enters the model as a continuous independent variable instead of a dichotomous variable**
    - Key dependent variables:
        - Quality of care: Ambulatory care sensitive (ACS) hospital admissions, ACS emergency department visits
        - Spending: 2019 Medicare **total** spending, geographically adjusted; excluding Part D

- Covariates:
    - Patient characteristics: race (white, black, Hispanic, other), age, sex, dual eligibility, CMS-HCC risk score from 2019
    - Physician characteristics: age (4-5 categories), sex
    - Practice characteristics: size (<=35, 36-100, 101-350, >350), ownership (hospital owned vs. private)

❖ Power Considerations

The primary data on physicians were collected prior to the planning of the current manuscript. A key research question related to the initial data collection was to examine whether altruism differs across physicians of different specialties, with the null hypothesis being that altruism of physicians does not differ by specialty. Our sample size of physicians in total and by specialty were sufficient to detect meaningful differences in altruism by specialty (see attached statistical plan submitted for Institutional Review Board approval for power calculations), which was examined in a previously published manuscript.

For the current manuscript, the focus is on examining differences in care quality and spending by physician altruism. Power calculations are not as helpful in this case as it is not possible to reliably estimate or predict *ex ante* what proportion of physicians we recruit would be classified as altruistic, or to purposefully recruit physicians in any given altruism category. The fact that our primary analyses yielded mostly statistically significant results suggest that we are sufficiently powered to detect meaningful differences in care quality and spending across patients of physicians with different altruism level, especially after controlling for the large number of physician and patient characteristics. It is possible that we do not have sufficient power to detect such differences when we restrict patients to smaller subgroups based on Medicaid eligibility, race or risk score.
