# Supplementary material for: Physician Altruism and Spending, Hospital Admissions, and Emergency Department Visits
Source: JAMA Health Forum. 2024 Oct 11;5(10):e243383. doi: 10.1001/jamahealthforum.2024.3383 (PMC11581536; doi:10.1001/jamahealthforum.2024.3383)
Supplement: Supplement 2. — eMethods 1. Experimental Instructions eMethods 2. Recruitment of Physician Subjects eMethods 3. Survey Questionnaire for Physicians eMethods 4. Survey Questionnaire for Medical Practice Leaders eMethods 5. Estimating Percentage Differences from Logistic Regression Results eMethods 6. Multiple Hypothesis Testing Adjustments Using the Holm-Bonferroni Method eMethods 7. Pre-registered Statistical Analysis Plan eFigure 1. Flowchart of analytic sample eFigure 2. Histogram of altruism parameter α, by altruism classification eFigure 3. Associations between altruism and care quality and spending across different altruism classifications eTable 1. Number of physicians and patients in each category of altruism classification, by model specification eTable 2. Associations between recruitment methods and physician altruism eTable 3. Associations between physician altruism and time spent on patient care eTable 4. Associations between physician altruism and time spent on patient care, classifying altruism by rejecting H0: α=0.5 vs. H1: α<0.5 using a one-sided t-test at the 1% level eTable 5. Associations between physician altruism and time spent on patient care, with α=0.5 as the cutoff for altruism eTable 6. Associations between physician altruism and time spent on patient care, with 1-α as the continuous measure of altruism [file jamahealthforum-e243383-s002.pdf]

Supplemental Online Content

Casalino LP, Kariv S, Markovits D, Fisman R, Li J. Physician altruism and spending, hospital admissions, and emergency department visits. *JAMA Health Forum*. 2024;5(10):e243383. doi:10.1001/jamahealthforum.2024.3383

eMethods 1. Experimental Instructions
eMethods 2. Recruitment of Physician Subjects
eMethods 3. Survey Questionnaire for Physicians
eMethods 4. Survey Questionnaire for Medical Practice Leaders
eMethods 5. Estimating Percentage Differences from Logistic Regression Results
eMethods 6. Multiple Hypothesis Testing Adjustments Using the Holm-Bonferroni Method
eMethods 7. Pre-registered Statistical Analysis Plan
eFigure 1. Flowchart of analytic sample
eFigure 2. Histogram of altruism parameter α, by altruism classification
eFigure 3. Associations between altruism and care quality and spending across different altruism classifications
eTable 1. Number of physicians and patients in each category of altruism classification, by model specification
eTable 2. Associations between recruitment methods and physician altruism
eTable 3. Associations between physician altruism and time spent on patient care
eTable 4. Associations between physician altruism and time spent on patient care, classifying altruism by rejecting H0: α=0.5 vs. H1: α<0.5 using a one-sided t-test at the 1% level
eTable 5. Associations between physician altruism and time spent on patient care, with α=0.5 as the cutoff for altruism
eTable 6. Associations between physician altruism and time spent on patient care, with 1-α as the continuous measure of altruism

This supplemental material has been provided by the authors to give readers additional information about their work.

© 2024 Casalino LP, et al. *JAMA Health Forum.*

eMethods 1. Experimental Instructions

In this experiment, you will make 25 decisions that share a common form. In each decision, you will be asked to allocate real money between yourself and another person. The other person will be chosen at random from the [Understanding America Study](https://uasdata.usc.edu) panel respondents. The Understanding America Study, or UAS is a panel at the University of Southern California of approximately 6,000 households representing the entire United States. More information about the UAS can be found at this link:

https://uasdata.usc.edu.

Clicking on the link opens a new window.

To further clarify, the UAS panel respondent is a real individual whose payoff from the experiment will depend on the amount of money you allocate to him or her, just as your payoff will depend on the amount of money you allocate to yourself

In each problem you will be asked to choose a point that is on a blue line like the one shown below.

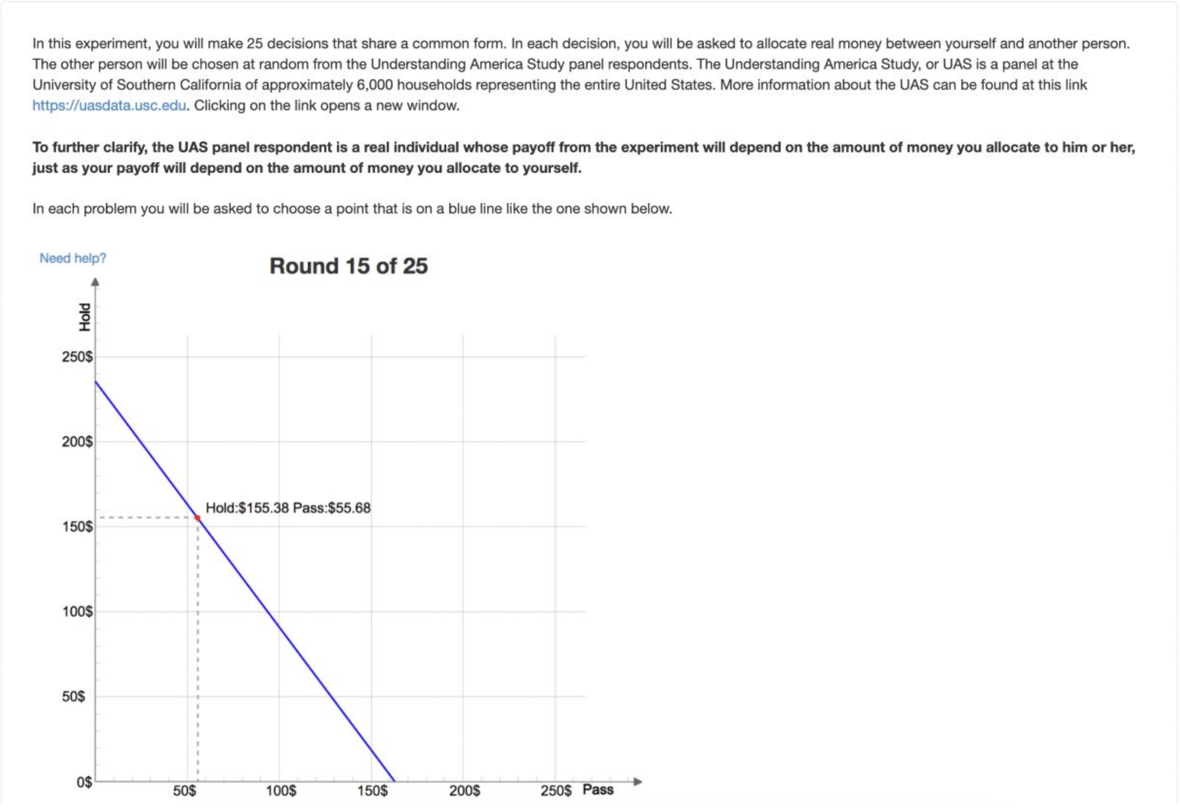

© 2024 Casalino LP, et al. *JAMA Health Forum.*

The line represents all the possible combinations of allocations you can choose to make. The vertical axis shows the number of experimental tokens you may allocate to yourself, or, hold. The horizontal axis shows the number of tokens you may allocate to the UAS panel respondent, or, pass. The tokens represent real money, and each token is equivalent to $2.5.

By selecting a point on the line, you are choosing how much you want to hold and how much you want to pass. For example, if you chose the point marked by the red ball, you would receive $155.38, and a randomly chosen UAS panel respondent will receive $55.68. You can select any point on the line.

Notice the trade-off between hold and pass. To increase the amount you hold, you have to reduce the amount you pass. To increase the amount you pass, you have to reduce the amount you hold.

To select your choice, use the mouse, or your finger if you are using a touch screen, to move the pointer on the computer screen to the option you wish to choose, and then click your mouse or trackpad once. A window like the one shown below will pop up.

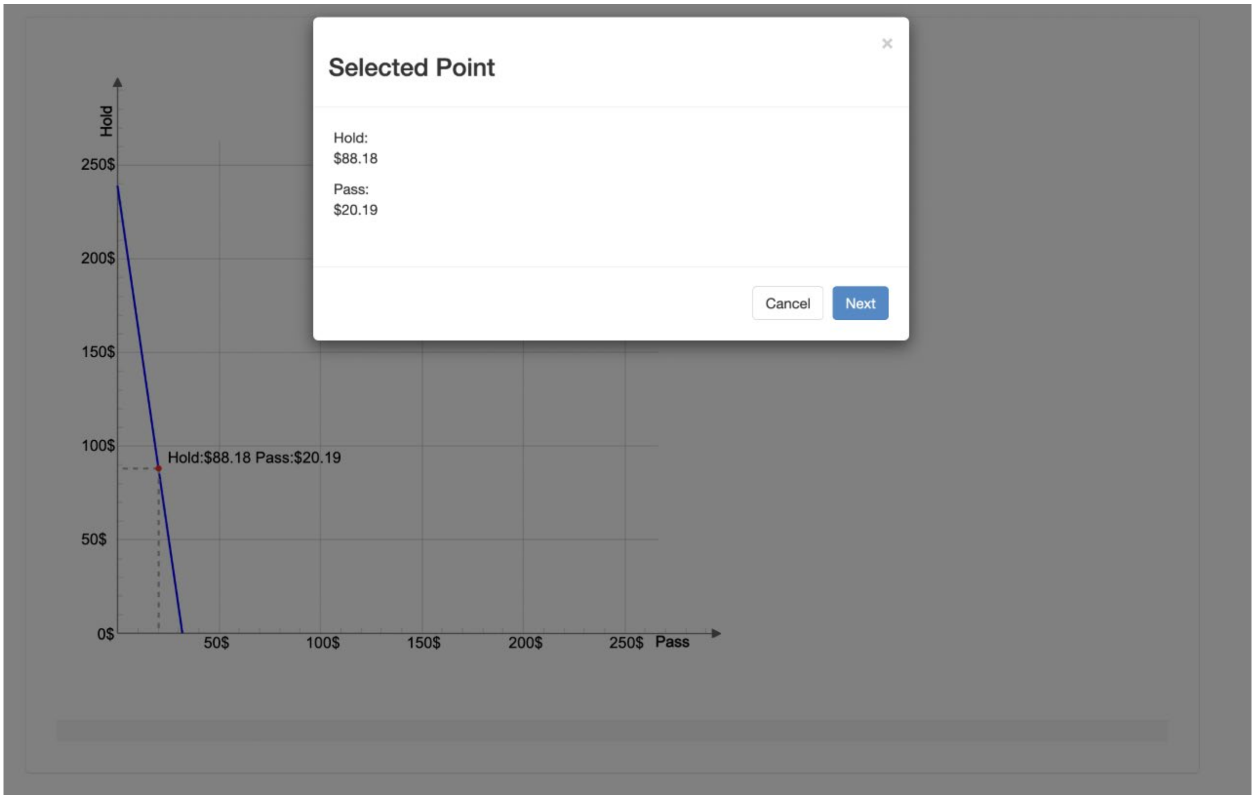

Press "Next" to confirm your choice or "Cancel" to revise your choice. You will not be able to revise your choice after pressing "Next".

© 2024 Casalino LP, et al. *JAMA Health Forum.*

After you click "Next", you will be moved to the next round, where you will be asked to make a choice in a different decision problem involving a different a line and different options. In total you will be asked to make 25 decisions.

Next, you will have two practice rounds. The choices you make in these two rounds will not affect how much money you will be paid. You can refer back to these instructions anytime you need to by clicking on the help link.

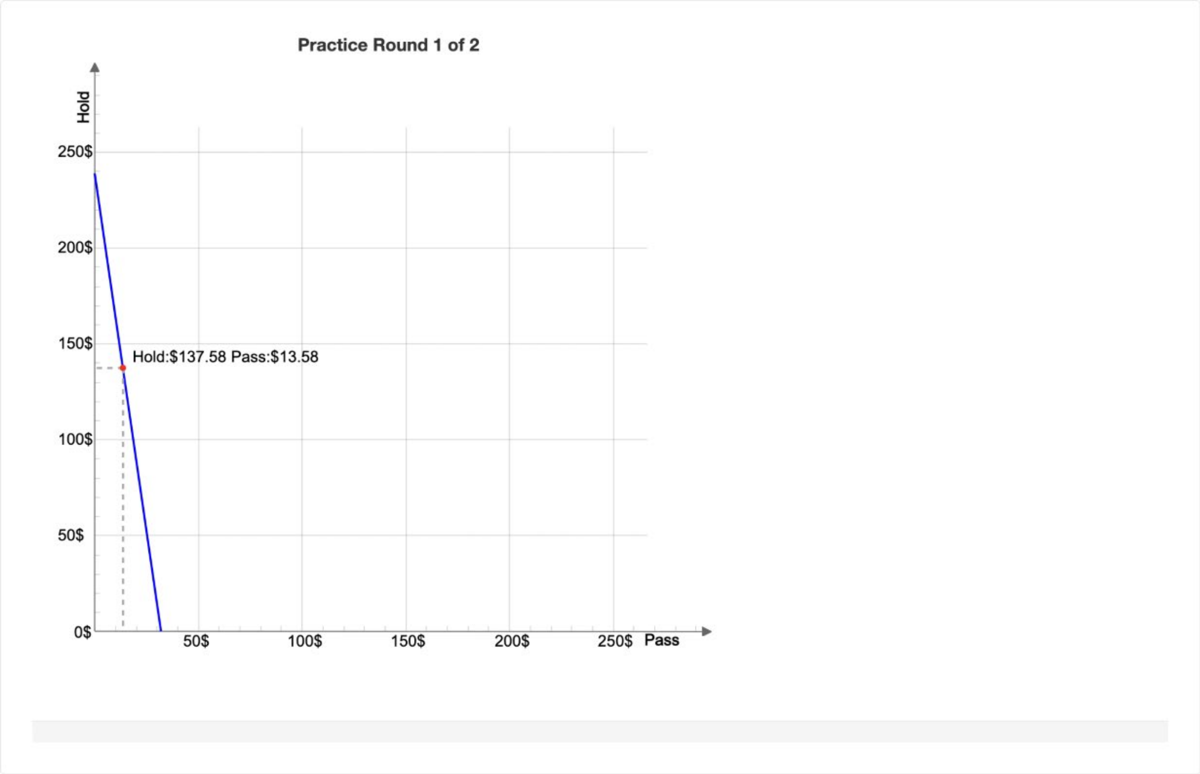

The instructions below explain how your payment will be determined based on the choices you make. After reading the instructions you will be ready to start making these choices.

Instructions

At the end of the survey the computer will randomly select one of the 25 problems for payment and you will be paid according to the choice you made in the selected problem.

Suppose that the computer selects problem 12 for payment. Let's say that in problem 12 you chose to hold $100 and pass $40.

If problem 12 is selected, you will be paid $100 dollars and a randomly selected UAS panel respondent will be paid $40 dollars.

Your payment will be emailed to you in a form of an Amazon gift card within five business days.

© 2024 Casalino LP, et al. *JAMA Health Forum.*

eMethods 2. Recruitment of Physician Subjects

We approached leaders of medical practices via email to ask them to ask their physicians to participate in the study. No database exists that provided a national listing of medical practices in the three specialties included in the study, so we identified practices in three ways:

1. Via practice leaders known by one of the authors (LC).
2. Via practice leaders known by one of the author's (LC) contacts.
3. Via web searches.

Contacts included, for example, the CEO of a major health insurance company, a former state health insurance commissioner, an executive with a major corporation that provides information to medical practices, and several current or former leaders of medical specialty societies leaders and of hospital systems. Twenty contacts recommended 49 medical practices.

Our objective was to include practices that varied in size, geographic region of the U.S., and whether or not they were part of an academic medical center. Eighty-seven practice leaders were invited to participate in the study; forty-three practices participated. Details of the survey sample are as follows.

Number of contacted and participating medical practices by medical specialty

	Internal Medicine	Family Medicine	Cardiology	Total
Contacted	30	8	49	87
Participating	18	3	22	43

Number of contacted and participating medical practices by method of recruitment and medical specialty

	Leader known to authors				Referred by authors' contacts				Web search			
	IM*	FM*	Card*	Total	IM	FM	Card	Total	IM	FM	Card	Total
Contacted	13	1	1	15	17	7	25	49	0	0	23	23
Participating	9	1	1	11	9	2	14	25	0	0	7	7

*IM: Internal Medicine; FM: Family Medicine. Card: Cardiology.

Number of contacted and participating medical practices by medical specialty and type of practice (private vs. hospital-based)

	Private				Hospital-based, including academic medical centers			
	IM	FM	Card	Total	IM	FM	Card	Total

© 2024 Casalino LP, et al. *JAMA Health Forum.*

Contacted	15	5	18	38	15	3	31	49
Participating	6	2	9	17	12	1	13	26

Number of contacted and participating medical practices by region

	Contacted	Participating
Northeast	23	15
Mid-Atlantic	10	4
South	12	4
Midwest	15	7
Southwest	15	9
West	12	4

Practice leaders were told that the purpose of the study was to measure physician altruism, but were asked not to mention this to their physicians. We provided an email message for leaders to use with their physicians. This email stated that:

> "I [the practice leader] am writing to let you know about an opportunity to participate in an innovative study designed by physicians at Cornell Medical College. The study focuses on tradeoffs that physicians make when allocating resources, and on the relationship between those tradeoffs and physician burnout. I would appreciate it if you would participate in this study. Participation will take **less than 15 minutes**, and you will receive **up to $250 ($150 on average)** by completing a very interesting experiment online."

We promised to send practice leaders results from the study as a whole and for their practice (not identifying individual physicians) in particular. We stated that at that time they could, if they wished, share the results concerning altruism.

Physicians were able to participate using a practice-specific weblink provided in the email. The website assigned each physician a unique code number once they provided their professional email address. The email address was later used to send payment associated with their decisions during the experiment in the form of an Amazon gift card.

At the beginning of the experiment, each physician responded to two questions via the website to determine their eligibility to participate (see Appendix 3). At the end of the experiment, each physician completed a short survey via the website that asked about demographic information (e.g. age, sex), average number of hours spent working at home on patient care, and other practice-related information. The questionnaire is included in Appendix 3.

Practice leaders completed a short survey by mail that provided basic information about their medical practice – e.g. on the number of physicians in their practice and type of practice. The practice leader survey questionnaire is included in Appendix 4.

© 2024 Casalino LP, et al. *JAMA Health Forum.*

eMethods 3. Survey Questionnaire for Physicians

NOTE: CAPITALIZED CONTENTS ARE INSTRUCTIONS FOR PROGRAMMERS ONLY. THEY WILL NOT APPEAR IN THE SURVEY INTERFACE.

SCREENING QUESTIONS

1. What is your highest medical degree?

    1] Doctor of Medicine (MD)

    2] Doctor of Osteopathic Medicine (DO)

    3] Other, including no medical degree

[IF 3] OTHER, NOTIFY THE PARTICIPANT THAT HE/SHE IS NOT ELIGIBLE FOR THE PROJECT.]

2. On average, approximately how many **hours** per **week** **(NOT per day)** are you scheduled to provide outpatient care? Please include only hours when you yourself are scheduled to see patients, not hours when you are supervising residents.

[IF ≤ 11 HOURS, NOTIFY THE PHYSICIAN THAT HE/SHE IS NOT ELIGIBLE FOR THE PROJECT.]

3. Do you practice in any of the specialties below?

    1] General internal medicine, not as a hospitalist

    2] Family medicine

    3]) Invasive or interventional cardiology (e.g. practice includes cardiac catheterizations, angioplasties, and stents)

    4] Noninvasive cardiology (but not primarily focused on cardiac imaging)

    5] None of the above

[IF NONE OF THE SPECIALTIES ABOVE, NOTIFY THE PHYSICIAN THAT HE/SHE IS NOT ELIGIBLE FOR THE PROJECT.]

© 2024 Casalino LP, et al. *JAMA Health Forum.*

4. Approximately how many years have you worked in the organization in which you currently work?

1] Less than one year
2] One to three years
3] More than three years

[IF LESS THAN ONE YEAR, NOTIFY THE PHYSICIAN THAT HE/SHE IS NOT ELIGIBLE FOR THE PROJECT.]

(GRAPHICAL DECISION PROBLEMS GO AFTER THE SCREENING QUETSIONS AND BEFORE THE MAIN SURVEY)

Next we will ask you a few standard survey questions. The survey should take no more than 5 minutes to complete. We really appreciate it that you answer these questions to the best of your knowledge, which is important to our study. Again, you may withdraw from the study at anytime.

Main Survey

1. What year did you graduate from medical school? (DROPDOWN MENU: 1960-2018)

2. What is your age?
1] 30 or under
2] 31-39
3] 40-49
4] 50-59
5] 60 or over

3. What is your gender?
1] Female
2] Male

For questions 4 to 6, think about a typical day when you are scheduled to see patients.

4. Approximately what time do you arrive at work? (DROPDOWN MENU: 6AM-5PM)

5. What time do you leave work? (DROPDOWN MENU: 10AM-11PM)

© 2024 Casalino LP, et al. *JAMA Health Forum.*

6. When you are seeing patients for office visits, approximately how many patients do you see, on average, in a typical three hour time period? (TYPE THE ANSWER)

7. On an average **day**, approximately how many **hours** do you spend at home, before and/or after work, on activities related to caring for your patients (e.g. communicating with patients and families by phone or email, working with the electronic medical record, reviewing test results)? Please round to the nearest half-hour – for example 3 or 3.5 hours.

8. What was your undergraduate major? Please select the closest one from the list. If you had more than one major, please select all of them.

Dropdown menu:

African and African American Studies

Anthropology

Applied Mathematics

Archaeology

Architectural Design

Art History

Astrophysics

Biochemistry

Biology

Biomedical Engineering

Business Administration

Chemical Engineering

Chemistry

Classics

Communications

Comparative Literature

Computer Science

Earth Science

Economics

Education

Electrical Engineering

English, Language, and Literature

Environmental Science

Feminist, Gender, and Sexuality Studies

History

© 2024 Casalino LP, et al. *JAMA Health Forum.*

International Relations

Journalism

Linguistics

Materials Science and Engineering

Mathematics

Mechanical Engineering

Molecular and Cellular Biology

Music

Neurobiology

Nursing

Philosophy

Physics

Political Science

Psychology

Religious Studies

Sociology

Statistics and Data Science

Theater, Dance, and Media

Visual Arts

Other, please specify________________________________

For questions 9 to 11, please choose the answer that best represents your experience:

9. I am able to choose the Registered Nurse/Licensed Vocational Nurse/Licensed Practical Nurse/Medical Assistant who works most closely with me.

Strongly disagree   Disagree   Neutral   Agree   Strongly agree

10. Overall, I am satisfied with my current job.

Strongly disagree   Disagree   Neutral   Agree   Strongly agree

11. My professional values are well aligned with those of my department leaders.

Strongly disagree   Disagree   Neutral   Agree   Strongly agree

© 2024 Casalino LP, et al. *JAMA Health Forum.*

12. Using your own definition of “burnout,” please circle one of the answers below:

1) I enjoy my work. I have no symptoms of burnout.
2) I am under stress, and don’t always have as much energy as I did, but I don’t feel burned out.
3) I am definitely burning out and have one or more symptoms of burnout, e.g., emotional exhaustion.
4) The symptoms of burnout that I am experiencing won’t go away. I think about work frustrations a lot.
5) I feel completely burned out. I am at the point where I may need to seek help.

13. Approximately what percentage of your patients are insured by Medicaid? (Please do not include patients with both Medicaid and Medicare insurance)

1) Under 25%
2) Between 25% and 50%
3) Between 50% and 75%
4) More than 75%

© 2024 Casalino LP, et al. *JAMA Health Forum.*

eMethods 4. Survey Questionnaire for Medical Practice Leaders

1. Please fill out the Table below. Please base your responses on the number of physicians in your medical practice, across all the practice's sites. Approximate numbers are fine.

	Number of physicians in your medical practice	Number to whom you sent requests to participate in the study
All physicians		
General internal medicine		
Family Practice		
Cardiology		

2. Please circle the choice that best explains the ownership of your medical practice.

Our physicians are employed by:

a) a nonprofit hospital
b) a for-profit hospital
c) an academic medical center
d) a medical practice owned by its physicians
e) a health insurance plan
f) a medical practice whose majority owner is a private equity firm
g) a publicly traded company
h) a community health center
g) other (please specify): ____

3. Does your medical practice contract to see patients via a medical foundation that is controlled by a hospital?

Yes ______ No _____

PLEASE CONTINUE TO PAGE 2

© 2024 Casalino LP, et al. *JAMA Health Forum.*

4. How does your medical practice pay its primary care physicians? Please circle the choice that best describes the way your primary care physicians are paid.

Our primary care physicians are paid primarily based on:

a) the volume of services that the physician provides

b) salary (not based mainly on the volume of services that the physician provides)

c) the physician's patient panel size

d) other: ____

5. How does your medical practice pay its cardiologists? Please circle the choice that best describes the way your cardiologists are paid.

Our cardiologists are paid primarily based on:

a) the volume of services that the physician provides

b) salary (not based mainly on the volume of services that the physician provides)

c) other: ____

© 2024 Casalino LP, et al. *JAMA Health Forum.*

eMethods 5. Estimating Percentage Differences from Logistic Regression Results

The following steps were taken to estimate adjusted percentage differences in the probability of having any ambulatory care sensitive admissions (ACSA) and the probability of having any ambulatory care sensitive emergency department (ACSED) visits between patients attributed to altruistic vs. non-altruistic physicians:

(1) Predict the probability of having any ACSA or any ACSED for each patient in the study sample **assuming they were all attributed to altruistic physicians** while keeping all other covariates unchanged, using the logistic regressions reported in Table 2;

(2) Predict the probability of having any ACSA or any ACSED for each patient in the study sample **assuming they were all attributed to NON-altruistic physicians** while keeping all other covariates unchanged, using the logistic regressions reported in Table 2;

(3) Take the difference between (1) and (2) for each patient, and obtain the mean of these differences across all patients;

(4) Divide the mean difference in (3) by the control mean of having any ACSA (0.026) or any ACSED (0.037) in Table 2 to obtain percentage differences in having any ACSA or any ACSED.

Steps (1)-(3) can be easily achieved by the -margins, dydx()- command in STATA to get the mean differences (called marginal effects). The marginal effect of being attributed to an altruistic physician was -0.010 for the probability of having any ACSA and -0.015 for the probability of having any ACSED. Hence:

Percentage diff. in Prob (ACSA) =

$$\frac{Mean\ Difference\ in\ predicted\ Prob(ACSA)}{Mean\ of\ any\ ACSA\ among\ patients\ of\ nonalruistic\ physicians} = \frac{-0.010}{0.026} = 38\%$$

Percentage diff. in Prob (ACSED) =

$$\frac{Mean\ Difference\ in\ predicted\ Prob(ACSED)}{Mean\ of\ any\ ACSED\ among\ patients\ of\ nonalruistic\ physicians} = \frac{-0.015}{0.037} = 41\%$$

© 2024 Casalino LP, et al. *JAMA Health Forum.*

eMethods 6. Multiple Hypothesis Testing Adjustments Using the Holm-Bonferroni Method

We performed adjustments for multiple hypothesis testing on our primary analyses results presented in Table 2 using the Holm-Bonferroni Method (Holm 1979). This method is more powerful than the alternative Bonferroni method which is commonly considered as overly conservative. The formula to calculate a revised alpha value using the Holm-Bonferroni Method is:

$$\frac{Target\ \alpha}{n - rank\ number\ of\ pair\ (by\ degree\ of\ significance) + 1}$$

Where $Target\ \alpha$ is the level of significance chosen under no adjustments (0.05), and n represents number of tests.

We preformed separate adjustments on the analyses on the full physician sample (three outcomes and three tests) and those on the mutually exclusive subsamples by physician specialty (three outcomes and six tests).

All results on the full physician sample in Table 2 that were significant ($p<0.05$) under no adjustments still reached statistical significance with the Holm-Bonferroni adjustment, i.e. we can reject the null hypothesis of no difference between patients treated by altruistic vs. non-altruistic physicians.

Among analyses on the subsample by specialty, all results on cardiologists that were significant ($p<0.05$) under no adjustments still reached statistical significance with the Holm-Bonferroni adjustment. None of the results on primary care physicians reached statistical significance with the Holm-Bonferroni adjustment.

References:

Holm, S., 1979. A simple sequentially rejective multiple test procedure. Scandinavian journal of statistics, pp.65-70.

© 2024 Casalino LP, et al. *JAMA Health Forum.*

eMethods 7. Pre-registered Statistical Analysis Plan

Note: This document is updated from the pre-specified statistical analysis plan (SAP) dated January 20, 2022 and registered on the Open Science Framework website: https://osf.io/75j8k. This current document reflects updates and changes in analyses made from the pre-specified SAP and are consistent with the submitted manuscript. Changes and updates are highlighted in bold. This document also includes a section on power discussions.

- ❖ Title: Physician altruism and care quality and spending among Medicare patients
- ❖ Funding: Physicians Foundation
- ❖ Research team: Lawrence Casalino, Jing Li, **Raymond Fisman, Shachar Kariv, Daniel Markovits**
- ❖ Research Question(s)
    1. Do more altruistic physicians as measured in a lab experiment provide better quality of care among Medicare patients?
    2. Do more altruistic physicians as measured in a lab experiment incur lower spending on care for Medicare patients?
    3. Does the relationship between physician altruism and care quality/spending differ by patient socioeconomic status and patient risk?

- ❖ Methods
    - ▪ Data source(s):
        - Primary data collected in 2019 on altruism of a nationwide sample of physicians in Internal Medicine, Cardiology and Family Medicine
        - 2019 Medicare Parts A & B claims data
    - ▪ Overview of analysis plan
        - Study sample (inclusion & exclusion restrictions):
            - ▪ **Physicians: 250 physicians with complete survey and experimental data, and who had at least three attributed Medicare patients in 2019** based on the attribution rule from CMS Merit-Based Incentive Payment System (by plurality of primary care claims)
            - ▪ Patients: **7,626** Medicare patients **aged 66 and above** who were continuously enrolled from January to December 2019 who were attributed to the **250** physicians included in the study.
        - Approach:
            - ▪ Multivariable regressions examining the relationships between altruism (independent variable) and quality of care and spending on care (dependent variables)
            - ▪ Stratified analysis by physician specialty (primary care vs. cardiology)
            - ▪ Stratified analysis by patient subgroup based on race (white vs. non-white), Medicaid eligibility, HCC top 20% risk score
            - ▪ Secondary analysis:

- Relationship between altruism and average time spend per patient visit, and time spent on patient care at home before or after work

- Variables:
  - Key independent variable: Altruism (Selfless vs. not selfless)
    - **Primary definition of selfless: cannot reject that α=0.5 vs. α<0.5, using one-sided t-test at 5% significance (this definition was adopted as it is the most stringent and most consistent with the inherent notion of altruism—physicians put unambiguously more weight on others' payoff than their own).**
    - **Alternative definitions for sensitivity analyses:**
      - **α<0.5 using point estimate only**
      - **α enters the model as a continuous independent variable instead of a dichotomous variable**
  - Key dependent variables:
    - Quality of care: Ambulatory care sensitive (ACS) hospital admissions, ACS emergency department visits
    - Spending: 2019 Medicare **total** spending, geographically adjusted; excluding Part D
  - Covariates:
    - Patient characteristics: race (white, black, Hispanic, other), age, sex, dual eligibility, CMS-HCC risk score from 2019
    - Physician characteristics: age (4-5 categories), sex
    - Practice characteristics: size (<=35, 36-100, 101-350, >350), ownership (hospital owned vs. private)

- **Power Considerations**

The primary data on physicians were collected prior to the planning of the current manuscript. A key research question related to the initial data collection was to examine whether altruism differs across physicians of different specialties, with the null hypothesis being that altruism of physicians does not differ by specialty. Our sample size of physicians in total and by specialty were sufficient to detect meaningful differences in altruism by specialty (see attached statistical plan submitted for Institutional Review Board approval for power calculations), which was examined in a previously published manuscript.
For the current manuscript, the focus is on examining differences in care quality and spending by physician altruism. Power calculations are not as helpful in this case as it is not possible to reliably estimate or predict *ex ante* what proportion of physicians we recruit would be classified as altruistic, or to purposefully recruit physicians in any given altruism category. The fact that our primary analyses yielded mostly statistically significant results suggest that we are sufficiently powered to detect meaningful differences in care quality and spending across patients of physicians with different altruism level, especially after controlling for the large number of physician and patient characteristics. It is possible that we do not have sufficient power to detect such differences when we restrict patients to smaller subgroups based on Medicaid eligibility, race or risk score.

© 2024 Casalino LP, et al. *JAMA Health Forum.*

eFigure 1. Flow Chart of Analytic Sample

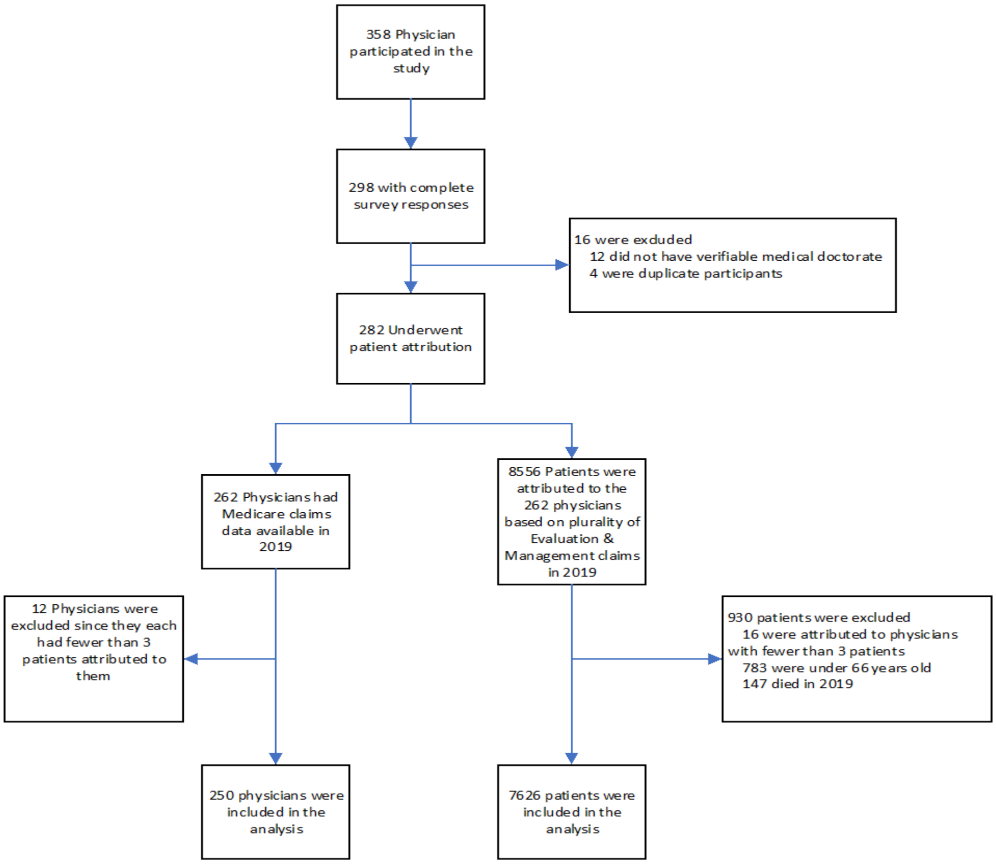

© 2024 Casalino LP, et al. *JAMA Health Forum.*

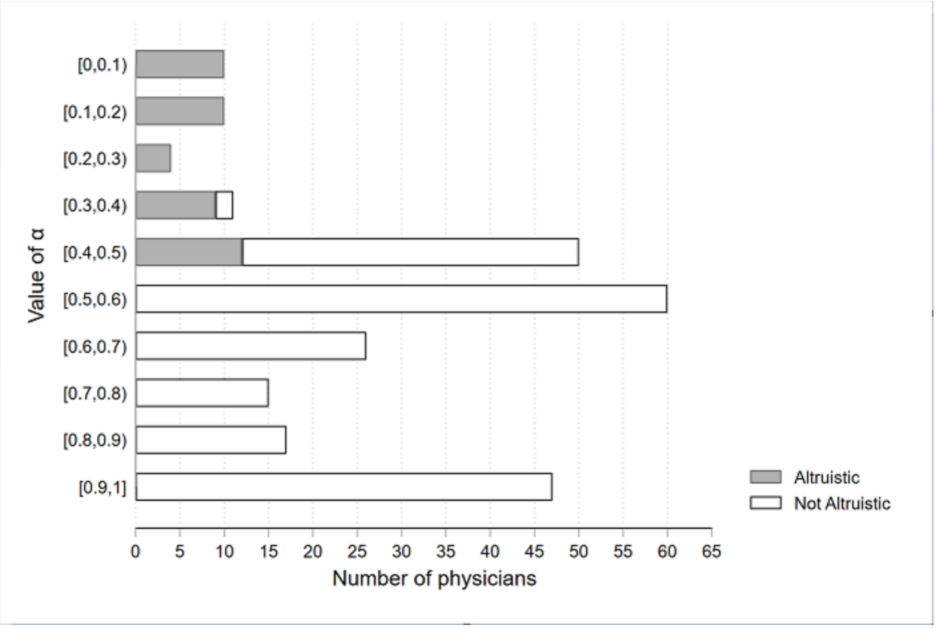

Title: eFigure 2. Histogram of altruism parameter α, by altruism classification

Notes: Frequency distribution in the altruism parameter α is shown separately for physicians classified as altruistic (in gray) and non-altruistic (in white). Physicians were classified as altruistic if we could reject that H_0: α=0.5 vs. H_1: α<0.5, using a one-sided *t*-test at 5% significance level, and non-altruistic otherwise. There is overlap in the two distributions between $\alpha = 0.3$ and $\alpha = 0.5$ because some of those classified as non-altruistic had large standard errors of their estimated α parameter such that we could not reject that their H_0: α=0.5 vs. H_1: α<0.5, even though the point estimate of α was smaller than 0.5.

© 2024 Casalino LP, et al. *JAMA Health Forum.*

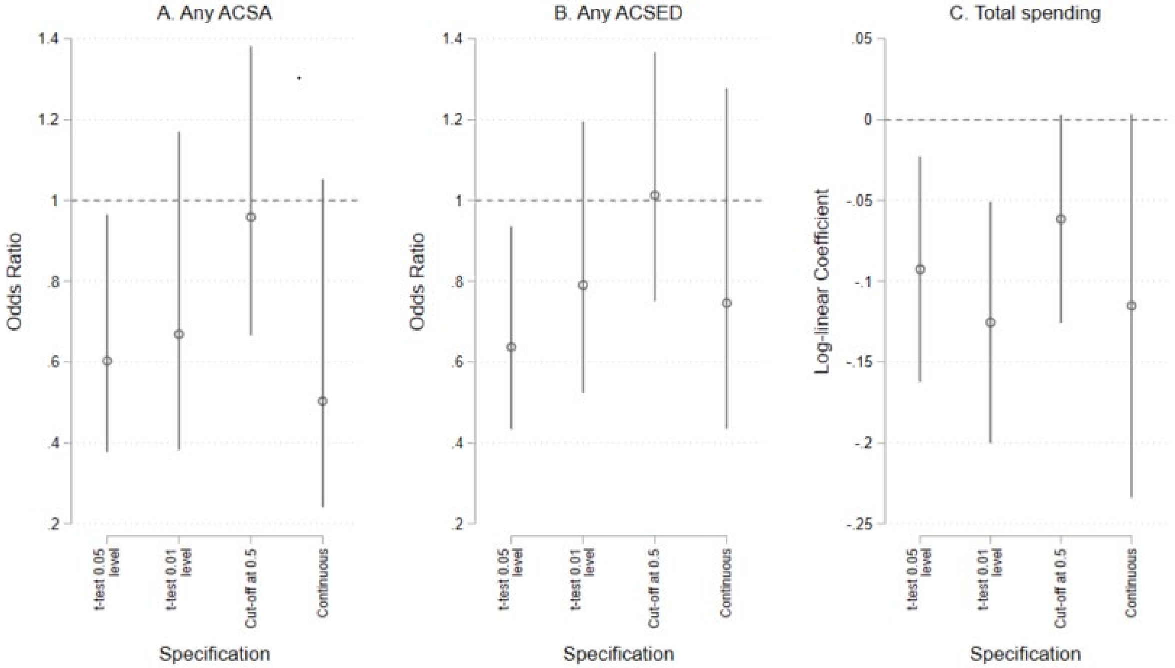

Title: eFigure 3. Associations between altruism and care quality and spending across different altruism classifications

Notes: ACSA=ambulatory care sensitive admission; ACSED = ambulatory care sensitive emergency department visit. Odds ratios (for ACSA and ACSED analyses) or coefficients (for spending analyses) on the altruism parameter are shown for three dependent variables, any ACSA (Panel A), any ACSED (Panel B) and logged total annual healthcare spending (Panel C), with bars indicating 95% confidence intervals. In each panel, results from four model specifications are shown, each with a different classification of altruism but otherwise identical dependent variables and control variables. The "T-test 0.05 level" specification was our main analysis, which classified a physician as altruistic if we could reject that H_0: $\alpha=0.5$ vs. H_1: $\alpha<0.5$, using a one-sided *t*-test at 5% significance level. The "T-test 0.01 level" specification classified a physician as altruistic if we could reject that H_0: $\alpha=0.5$ vs. H_1: $\alpha<0.5$, using a one-sided *t*-test at 1% significance level. The "cut-off at 0.5" specification classified physicians as altruistic if their point estimate of α was below 0.5, and non-altruistic otherwise. The "continuous" specification used the raw continuous α as the main independent variable. Regressions in Panel A and Panel B were logistic models, and regressions in Panel C were log-linear models. All regressions controlled for the physician and patient characteristics in Table 1, with standard errors clustered by physician.

© 2024 Casalino LP, et al. *JAMA Health Forum.*

eTable 1. Number of physicians and patients in each category of altruism classification, by classification methods

Model specification	Classification method	Altruism category	Category definition	No. (%) of physicians (N=250)	No. (%) of attributed patients (N=7,626)
1 (Main specification)	Statistical test	Altruistic	Cannot reject that α=0.5 vs. α<0.5, one-sided test at 5% significance	45 (18.0)	1,599 (21.0)
		Not Altruistic	Reject that α=0.5 vs. α<0.5, one-sided test at 5% significance	205 (82.0)	6,027 (79.0)
2	Statistical test	Altruistic	Cannot reject that α=0.5 vs. α<0.5, one-sided test at 1% significance	38 (15.2)	1,399 (18.4)
		Not Altruistic	Reject that α=0.5 vs. α<0.5, one-sided test at 1% significance	212 (84.8)	6,227 (81.7)
3	Cutoff value	Altruistic	α<0.5	85 (34.0)	2,643 (34.7)
		Not Altruistic	$\alpha \geq 0.5$	165 (66.0)	4,983 (65.3)
4	Continuous	N/A	Raw value of α	250 (100.0)	7,626 (100.0)

© 2024 Casalino LP, et al. *JAMA Health Forum.*

eTable 2. Associations between recruitment methods and physician altruism

	(1) Altruistic (statistical test at 5% level)	(2) Altruistic (statistical test at 1% level)	(3) Altruistic (if α less than 0.5)	(4) α
(Omitted: author's contact)	-	-	-	-
Referred by author's contact	0.12	0.09	0.01	-0.05
95% CI	[0.00, 0.24]	[-0.02, 0.21]	[-0.14, 0.17]	[-0.14, 0.03]
p-value	(0.052)	(0.117)	(0.895)	(0.196)
Web search	-0.08	-0.09	-0.02	0.02
95% CI	[-0.37, 0.21]	[-0.35, 0.18]	[-0.38, 0.34]	[-0.18, 0.21]
p-value	(0.578)	(0.532)	(0.904)	(0.862)
Controls	Yes	Yes	Yes	Yes
Mean of Dep Var	0.18	0.15	0.34	0.60
Number of Observations	250	250	250	250

Notes: The dependent variable in Column (1) is an indicator of whether the physician was altruistic using our primary definition of altruism, same as Specification (1) in Table S1. The dependent variable in Column (2) is an indicator that equals 1 if we can reject that H_0: α=0.5 vs. H_1: α<0.5 using a one-sided *t*-test at the 1% level. The dependent variable in Column (3) is an indicator that equals 1 if α< 0.5. The dependent variable in Column (4) is the raw continuous α (where a higher value indicates lower altruism). A higher coefficient in Column (1)- (3) indicates higher altruism, whereas a higher coefficient in Column (4) indicates lower altruism. All regressions controlled for cardiology specialty, ownership (private vs. hospital-owned), their interactions, physician age category, practice region and practice size. Standard errors in parenthesis.

© 2024 Casalino LP, et al. *JAMA Health Forum.*

eTable 3. Associations between physician altruism and time spent on patient care

Outcome	Unadjusted mean, Patients attributed to non-altruistic physicians (N=205)	Unadjusted mean, Patient attributed to altruistic physicians (N=45)	Adjusted association (Odds ratio for spending one hour or more on patient care and linear coefficient for number of patients seen)	95% confidence interval	P Value
Probability of spending one hour or more on patient care at home per day					
All Physicians	0.561	0.689	2.034	[0.965,4.287]	0.062
Primary Care	0.612	0.688	1.638	[0.676,3.968]	0.274
Cardiology	0.465	0.692	3.720	[0.890,15.554]	0.072
Number of patients seen in a three-hour period					
All Physicians	8.904	8.567	-0.450	[-1.221,0.320]	0.251
Primary Care	8.465	7.781	-0.939	[-1.577,-0.301]	0.004
Cardiology	9.732	10.500	0.627	[-1.637,2.891]	0.583

Notes: Physicians were classified as altruistic if we could reject that H_0: $\alpha=0.5$ vs. H_1: $\alpha<0.5$, using a one-sided t-test at 5% significance level, and non-altruistic otherwise. Adjusted associations were estimated using multivariable regressions (logistic regressions for probability of spending one hour or more on patient care at home and linear model for number of patients seen in a three-hour period) that controlled for all physician characteristics in Table 1. Each row reports results from a separate regression for all physicians or by specialty (primary care and cardiology). Heteroskedasticity robust standard errors were used.

© 2024 Casalino LP, et al. *JAMA Health Forum.*

eTable 4. Associations between physician altruism and time spent on patient care, classifying altruism by rejecting H_0: α=0.5 vs. H_1: α<0.5 **using a one-sided *t*-test at the 1% level**

Panel A			
Dependent variable	Spending one hour or more on patient care at home		
	(1) All	(2) Primary Care	(3) Cardiology
Indicator for α<0.5 at the 1% significance level	2.163	2.312	3.217
95% CI	[0.973,4.811]	[0.846,6.316]	[0.744,13.913]
p-value	(0.059)	(0.102)	(0.118)
Mean of Dep Var	0.58	0.63	0.50
Number of Observations	250	166	84

Panel B			
Dependent variable	Number of patients seen in 3 hours		
	(1) All	(2) Primary Care	(3) Cardiology
Indicator for α<0.5 at the 1% significance level	-0.219	-0.673	0.695
95% CI	[-1.057,0.619]	[-1.285,-0.060]	[-1.738,3.128]
p-value	(0.607)	(0.032)	(0.571)
Mean of Dep Var	8.84	8.33	9.85
Number of Observations	250	166	84

Notes: Physicians were classified as altruistic if we could reject that H_0: α=0.5 vs. H_1: α<0.5 , using a one-sided t-test at 1% significance level, and non-altruistic otherwise. Adjusted associations were estimated using multivariable regressions (logistic regressions for probability of spending one hour or more on patient care at home and linear model for number of patients seen in a three-hour period) that controlled for all physician characteristics in Table 1. Odds ratios are reported in Panel A and regression coefficients are reported in Panel B. Each column reports results from a separate regression for all physicians or by specialty (primary care and cardiology). Heteroskedasticity robust standard errors were used.

© 2024 Casalino LP, et al. *JAMA Health Forum.*

eTable 5. Associations between physician altruism and time spent on patient care, with α=0.5 as the cutoff for altruism

Panel A			
Dependent variable	Spending one hour or more on patient care at home		
	(1) All	(2) Primary Care	(3) Cardiology
$\alpha<0.5$	1.603	1.185	6.260
95% CI	[0.885,2.902]	[0.605,2.324]	[1.689,23.201]
p-value	(0.119)	(0.621)	(0.006)
Mean of Dep Var	0.58	0.63	0.50
Number of Observations	250	166	84

Panel B			
Dependent variable	Number of patients seen in 3 hours		
	(1) All	(2) Primary Care	(3) Cardiology
$\alpha<0.5$	0.087	-0.234	0.965
95% CI	[-0.555,0.728]	[-0.886,0.418]	[-0.673,2.603]
p-value	(0.791)	(0.479)	(0.244)
Mean of Dep Var	8.84	8.33	9.85
Number of Observations	250	166	84

Notes: Physicians were classified as altruistic if α<0.5, and non-altruistic otherwise. Adjusted associations were estimated using multivariable regressions (logistic regressions for probability of spending one hour or more on patient care at home and linear model for number of patients seen in a three-hour period) that controlled for all physician characteristics in Table 1. Each column reports results from a separate regression for all physicians or by specialty (primary care and cardiology). Heteroskedasticity robust standard errors were used.

© 2024 Casalino LP, et al. *JAMA Health Forum.*

eTable 6. Associations between physician altruism and time spent on patient care, with 1-α as the continuous measure of altruism

Panel A			
Dependent variable	Spending one hour or more on patient care at home		
	(1)<br>All	(2)<br>Primary Care	(3)<br>Cardiology
1- α	1.948	1.276	6.470
95% CI	[0.671,5.658]	[0.333,4.893]	[0.875,47.833]
p-value	(0.220)	(0.723)	(0.067)
Mean of Dep Var	0.58	0.63	0.50
Number of Observations	250	166	84

Panel B			
Dependent variable	Number of patients seen in 3 hours		
	(1)<br>All	(2)<br>Primary Care	(3)<br>Cardiology
1- α	0.492	-0.266	0.950
95% CI	[-0.739,1.724]	[-1.566,1.034]	[-1.600,3.501]
p-value	(0.432)	(0.686)	(0.460)
Mean of Dep Var	8.84	8.33	9.85
Number of Observations	250	166	84

Notes: 1-α was used as the main independent variable for altruism. Adjusted associations were estimated using multivariable regressions (logistic regressions for probability of spending one hour or more on patient care at home and linear model for number of patients seen in a three-hour period) that controlled for all physician characteristics in Table 1. Each column reports results from a separate regression for all physicians or by specialty (primary care and cardiology). Heteroskedasticity robust standard errors were used.

© 2024 Casalino LP, et al. *JAMA Health Forum.*
