# Supplementary material for: Physician Altruism and Spending, Hospital Admissions, and Emergency Department Visits
Source: JAMA Health Forum. 2024 Oct 11;5(10):e243383. doi: 10.1001/jamahealthforum.2024.3383 (PMC11581536; doi:10.1001/jamahealthforum.2024.3383)
Supplement: Supplement 3. — Data Sharing Statement [file jamahealthforum-e243383-s003.pdf]

Data Sharing Statement

Casalino. Physician Altruism and Spending, Hospital Admissions, and Emergency Department Visits. *JAMA Health Forum*. Published October 11, 2024.
doi:10.1001/jamahealthforum.2024.3383

Data
Data available: No

Additional Information
Explanation for why data not available: Our final analytic data file is derived partly from proprietary Medicare claims data which must be accessed via a Data Use Agreement with Centers for Medicare and Medicaid Services and is not available to share.
